# Healthy Movement Leads to Emotional Connection: Development of the Movement Poomasi “Wello!” Application Based on Digital Psychosocial Touch—A Mixed-Methods Study

**DOI:** 10.3390/healthcare13172157

**Published:** 2025-08-29

**Authors:** Suyoung Hwang, Hyunmoon Kim, Eun-Surk Yi

**Affiliations:** 1 Department of Exercise Rehabilitation & Welfare, Gachon University, Incheon 21936, Republic of Korea; harriett0059@gmail.com; 2 Department of IT Policy and Management, Soongsil University, 369, Sangdo-ro, Dongjak-gu, Seoul 06978, Republic of Korea

**Keywords:** digital healthcare for seniors, usability and accessibility, hybrid exercise models, social engagement in aging, technology adoption in older adults

## Abstract

**Background/Objective:** The global acceleration of population aging presents profound challenges to the physical, psychological, and social well-being of older adults. As traditional exercise programs face limitations in accessibility, personalization, and sustained social support, there is a critical need for innovative, inclusive, and community-integrated digital movement solutions. This study aimed to develop and evaluate Movement Poomasi, a hybrid digital healthcare application designed to promote physical activity, improve digital accessibility, and strengthen social connectedness among older adults. **Methods:** From March 2023 to November 2023, Movement Poomasi was developed through an iterative user-centered design process involving domain experts in physical therapy and sports psychology. In this study, the term UI/UX—short for user interface and user experience—refers to the overall design and interaction framework of the application, encompassing visual layout, navigation flow, accessibility features, and user engagement optimization tailored to older adults’ sensory, cognitive, and motor characteristics. The application integrates adaptive exercise modules, senior-optimized UI/UX, voice-assisted navigation, and peer-interaction features to enable both home-based and in-person movement engagement. A two-phase usability validation was conducted. A 4-week pilot test with 15 older adults assessed the prototype, followed by a formal 6-week study with 50 participants (≥65 years), stratified by digital literacy and activity background. Quantitative metrics—movement completion rates, session duration, and engagement with social features—were analyzed alongside semi-structured interviews. Statistical analysis included ANOVA and regression to examine usability and engagement outcomes. The application has continued iterative testing and refinement until May 2025, and it is scheduled for re-launch under the name Wello! in August 2025. **Results:** Post-implementation UI refinements significantly increased navigation success rates (from 68% to 87%, *p* = 0.042). ANOVA revealed that movement selection and peer-interaction tasks posed greater cognitive load (*p* < 0.01). A strong positive correlation was found between digital literacy and task performance (*r* = 0.68, *p* < 0.05). Weekly participation increased by 38%, with 81% of participants reporting enhanced social connectedness through group challenges and hybrid peer-led meetups. Despite high satisfaction scores (mean 4.6 ± 0.4), usability challenges remained among low-literacy users, indicating the need for further interface simplification. **Conclusions:** The findings underscore the potential of hybrid digital platforms tailored to older adults’ physical, cognitive, and social needs. Movement Poomasi demonstrates scalable feasibility and contributes to reducing the digital divide while fostering active aging. Future directions include AI-assisted onboarding, adaptive tutorials, and expanded integration with community care ecosystems to enhance long-term engagement and inclusivity.

## 1. Introduction

The global demographic landscape is rapidly shifting, with countries such as South Korea facing some of the fastest aging transitions. By 2025, older adults will comprise over 20% of South Korea’s population, marking it as a super-aged society [1]. Among these challenges, the decline in physical activity levels among older adults is a critical issue that contributes to deteriorating health, rising healthcare costs, and greater social isolation [2]. Physical inactivity is closely linked to chronic illnesses, cognitive decline, and depression [3], creating a cycle of social withdrawal and worsening of well-being [4]. Promoting regular physical activity has proven benefits, including enhanced cognitive function, psychological resilience, and improved quality of life [5,6,7]. As mental health concerns increase in aging populations, physical movement is a critical preventive strategy [8,9,10]. Beyond individual health, physical activity fosters social connectedness, which is increasingly important as older adults face diminished peer interactions. Structured movement programs can offer community engagement and peer support, combating loneliness and enhancing motivation [11,12].

Despite these benefits, older adults encounter barriers to active living, such as physical discomfort, fear of injury, and cognitive limitations [13,14,15]. Social isolation further reduces motivation, creating a need for new and accessible approaches [16]. The COVID-19 pandemic worsened these barriers, limiting access to in-person programs and increasing inactivity and isolation [17,18]. However, it also accelerated the adoption of digital healthcare, highlighting new opportunities for remote engagement [19,20].

Digital movement solutions, such as AI-powered mobile applications, offer personalized activity plans, real-time feedback, and community features [21,22]. However, many existing platforms overlook the psychological and social needs of older users and lack user-friendly interfaces for those with limited digital literacy [23,24,25]. To enhance engagement, digital interventions must integrate personalized guidance, usability, and peer support [26,27,28,29].

This study introduces Movement Poomasi, a community-based digital platform inspired by South Korea’s traditional “Poomasi” mutual aid system [30]. The platform aims to improve physical activity by integrating peer reinforcement, tailored guidance, and intuitive design. This study presents the development and evaluation of Movement Poomasi, offering empirical insights into how digital tools can support long-term movement adherence, emotional well-being, and social connections in aging populations.

Unlike conventional health applications targeting older adults, Movement Poomasi integrates Korea’s traditional community-sharing culture (“Poomasi”), a hybrid participation model combining face-to-face and digital interaction and an age-friendly adaptive UI/UX. This novel integration positions our approach as a culturally grounded and technically innovative solution to bridging digital divides in aging societies.

## 2. Methods

### 2.1. Development and Design of the Movement Poomasi “Wello!” Application

The development of Movement Poomasi “*Wello!*” was grounded in a user-centered digital health design strategy aimed at promoting physical activity, fostering social connectivity, and enhancing overall well-being among older adults. Anchored in a structured, evidence-informed framework, the development process unfolded through four iterative stages: user-needs assessment, expert consultation, prototype refinement, and real-world usability evaluation.

**Figure 1:**
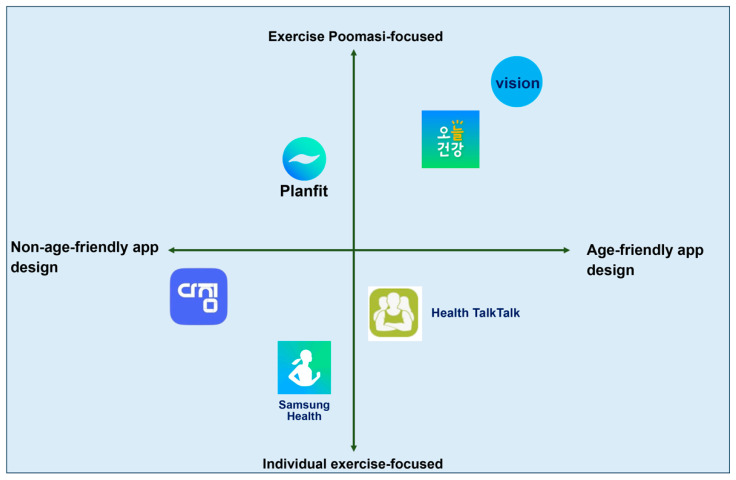
Competitive positioning of healthcare applications. *The vertical axis represents the focus on “Exercise Poomasi” versus individual exercise, while the horizontal axis contrasts non-age-friendly and age-friendly app designs. Applications plotted include Planfit, Samsung Health, Health TalkTalk, Vision, and two Korean healthcare apps (Dazim [다짐] and Oneul Geongang [오늘 건강]), which are widely used in South Korea to support fitness and wellness management*.

To position the application within the broader digital health ecosystem and identify critical gaps in existing services, a comparative benchmarking analysis was conducted (Figure 1, Table 1). This analysis revealed that many current senior-targeted health and fitness platforms fall short in three essential domains: integration of community-based support, flexibility in hybrid (in-person and digital) participation, and interface designs attuned to the cognitive and sensory needs of aging users. These shortcomings provided a foundational rationale for a novel, inclusive design approach.

Informed by this gap analysis, Movement Poomasi “*Wello!*” was deliberately crafted to overcome these limitations through three core design pillars: (1) embedded peer interaction and community-driven features; (2) seamless transitions between virtual and physical exercise formats; and (3) a UI/UX architecture specifically optimized for older adults, featuring simplified navigation, large-format visual elements, voice assistance, and intuitive user flows.

This foundational design philosophy was operationalized through an interdisciplinary collaboration that integrated insights from movement physiology, gerontology, psychology, and digital health. The result is a platform that not only addresses the functional dimensions of senior exercise participation but also aligns with their psychosocial and technological realities, thereby enhancing engagement, accessibility, and sustainability. The application underwent iterative testing and refinement through May 2025, and it is scheduled for re-launch under the name *Wello!* in August 2025.

### 2.2. User-Needs Assessment

A mixed-methods approach was used to identify the key barriers and facilitators of movement among older adults. A survey of 150 participants aged ≥ 65 years, recruited from senior centers and health programs, assessed their movement habits, digital literacy, and motivations. From this group, 30 individuals participated in in-depth interviews and focus groups to further explore the usability and accessibility challenges.

The interview transcripts were subjected to qualitative thematic analysis, which informed the functional and design priorities of the application.

### 2.3. Expert Collaboration and Interactive Development

A multidisciplinary team guided the development process. A movement physiologist designed safe, progressive routines; a welfare specialist advised on motivation strategies; a digital health expert ensured technical usability; and psychology researchers contributed to peer-support mechanisms. The process was divided into three phases:

1. Prototype Development: Initial wireframes incorporated custom movement plans, educational content, and peer engagement features.
2. Expert Review: Experts refined the prototype, enhancing accessibility through voice assistance, simplified navigation, and UI adjustments.
3. Usability Trials: Twenty older adults tested the app for four weeks. Feedback prompted final updates, including high-contrast text, onboarding tutorials, and simplified navigation.

**Figure 2:**
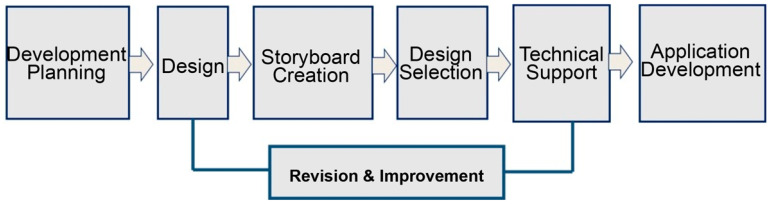
Development process: *Iterative development pipeline for Movement Poomasi “Wello!”, showing sequential phases and revision loops for continuous refinement*.

To ensure sustained physical activity, social connectivity, and accessibility for older adults, a user-centered iterative design strategy was implemented throughout the development process. This approach included benchmarking against leading digital health platforms and continuous refinement based on user and expert feedback. The overall development flow—including UI/UX design, system architecture, user feedback integration, and testing procedures—is illustrated in Figure 2.

### 2.4. Movement Program Structure and Qualitative Inquiry

The exercise program was designed to accommodate older adults’ physical needs and promote safe, sustainable home-based engagement. The structure included three main phases (warm-up, main training, and cool-down), each with distinct physiological and psychological objectives. The full program was integrated into the Movement Poomasi application and delivered through interactive audiovisual guidance.

In addition to traditional circuit and part-specific training, the program incorporated the ACPT–Fascial Circulation Exercise (ACPT-FCE) protocol: a movement-based intervention co-developed by Yeji Choi, a certified movement specialist, and Dr. Suyoung Hwang, a research professor specializing in sports psychology. This protocol is currently being systematized into a formal instructor certification curriculum and will be introduced in an upcoming peer-reviewed publication on the hybrid Movement Poomasi model. The ACPT-FCE method emphasizes neuromyofascial activation through rhythmic, flow-based movement patterns designed to enhance circulation, balance, and proprioceptive awareness. It further integrates breathing and sensory alignment techniques aimed at improving both physical mobility and emotional self-regulation in older adults.

The exercises were tiered by intensity level and supported by visual/audio guides to ensure accessibility for users with musculoskeletal limitations or low digital literacy. Table 2 outlines the revised structure of the program and its functional domains.

To gain in-depth insights into user experience and program adaptation, a qualitative evaluation was also conducted alongside usability testing. A semi-structured interview guide was developed based on the prior literature on digital health adoption in older adults, focusing on perceived usefulness, barriers to engagement, emotional resonance, and peer interactions. The guide was reviewed by three domain experts (in gerontology, rehabilitation, and digital design) to ensure relevance and clarity.

All interviews and focus groups were conducted in person by trained facilitators in community centers or senior activity hubs. Sessions were audio-recorded with participant consent and transcribed verbatim. The interview duration ranged from 45 to 70 min per session. Thematic analysis was performed using an inductive coding approach, allowing themes to emerge directly from participant narratives without a pre-existing framework.

Two independent coders (one psychologist and one public health researcher) analyzed the transcripts separately using NVivo 12 software. The initial codes were compared, and disagreements were resolved through discussion and reconciliation meetings. The process continued until thematic saturation was reached, which occurred after approximately 25 interviews, ensuring that no new major themes emerged.

To enhance the trustworthiness of the qualitative findings, the study followed established strategies including triangulation (data source and analyst), member checking with a subset of participants (*n* = 8), and audit trail documentation. Illustrative quotes were selected from across the participant profiles to support theme interpretation and are presented in the Results section to provide contextual depth.

### 2.5. Implementation and Usability Testing

To evaluate the real-world feasibility and engagement dynamics of the Movement Poomasi application, a two-phase usability validation strategy was implemented, adhering to both formative and summative evaluation principles.

In the initial phase, 15 older adults (≥65 years), purposively selected to reflect diverse levels of digital familiarity, participated in a 4-week pilot usability trial designed for early prototype refinement. Participants engaged with the application in a home-based setting and provided feedback on key dimensions such as interface navigation, clarity, and overall user experience. Semi-structured face-to-face interviews lasting 30–45 min were conducted post-trial, audio-recorded with consent, and transcribed verbatim. Thematic analysis of these transcripts informed critical UI/UX optimizations during the design iteration process.

In the subsequent main phase, a 6-week formal usability study was conducted with a broader cohort of 50 older adults, stratified by digital literacy level (experienced vs. limited exposure) and physical activity background (sedentary vs. active). The participants completed structured home-based movement sessions using the finalized application. Behavioral engagement was quantitatively monitored through three key indicators:

(1) Movement completion rate, measuring the percentage of completed weekly sessions; (2) session duration, reflecting the average time spent per exercise session; and (3) feature-specific engagement patterns, which tracked interactive usage frequencies, drop-off points, and the adoption of core functionalities such as peer challenges and onboarding tutorials.

To enrich these quantitative findings, an additional subsample of 15 participants from the main study engaged in follow-up semi-structured interviews. These interviews explored subjective experiences, navigation challenges, perceived usability, and motivational barriers to sustained engagement. Each interview lasted 30–45 min, was audio-recorded with consent, and was thematically analyzed.

This layered mixed-methods approach offered a comprehensive perspective on both objective interaction data and users’ perceptual insights, allowing for iterative refinement and robust evaluation of the application’s usability, accessibility, and user-centered design effectiveness.

### 2.6. Evaluation Methods

The evaluation of Movement Poomasi was conducted using an integrated mixed-methods framework, comprising both quantitative and qualitative components to ensure a comprehensive understanding of usability, accessibility, and health-related outcomes.

1. Quantitative Assessment:
  A 28-item user satisfaction questionnaire was administered using a 5-point Likert scale (1 = strongly disagree to 5 = strongly agree). The questionnaire was developed to comprehensively evaluate participants’ post-intervention experience with the Movement Poomasi application and consisted of five domains: navigation ease (6 items), visual clarity (5 items), motivational support (6 items), exercise guidance (6 items), and perceived benefit (5 items). Navigation ease assessed the clarity of the menu structure, the ease of locating desired functions, and the responsiveness of the interface. Visual clarity evaluated the readability of text, appropriateness of font size, color contrast, and recognizability of icons. Motivational support measured perceived encouragement, goal-setting functions, and reminder features designed to promote continued engagement. Exercise guidance evaluated the clarity of movement instructions, the appropriateness of exercise difficulty, and the inclusion of safety cues. Perceived benefit assessed the extent to which the application enhanced physical activity, social connectedness, and overall well-being.

Functional outcomes additionally included pre- and post-intervention self-reports of physical activity (weekly minutes), psychological well-being (mood, motivation, social connectedness), and digital self-efficacy. System logs were analyzed to capture participation rates, session frequency, and usage patterns of key features. Data collection for the satisfaction survey was conducted from March 2023 to May 2023. Statistical analysis was performed using SPSS v26. Paired *t*-tests assessed changes in physical activity and well-being, while ANOVA and multiple regression analyses examined the predictive effects of user variables such as digital literacy and baseline activity levels. Statistical significance was set at *p* < 0.05.

2. Qualitative Assessment:
  Thematic analysis of interview transcripts and open-ended survey responses was guided by Braun and Clarke’s six-step framework [31]. Coding focused on usability challenges, interface difficulties, and motivational factors. Findings were triangulated with behavioral data and narrative responses to ensure validity. Member checking with five participants and peer debriefing among the research team enhanced the credibility and analytical rigor.

This comprehensive evaluation elucidated how older adults interacted with the app, the facilitators of and barriers to their use, and the extent to which the app supported sustained engagement and psychosocial benefits.

### 2.7. Ethical Considerations

This study adhered to ethical research standards for human participants. Approval was obtained from the Institutional Review Board (IRB) of Gachon University (IRB No. 1044396-202305-HR-084-01). Informed consent was obtained from all participants prior to data collection. Additionally, all participant data were anonymized, and confidentiality was maintained in accordance with research ethics guidelines.

Clinical trial number: not applicable.

## 3. Results

### 3.1. Thematic Findings from User-Needs Assessment

Thematic analysis revealed three core barriers that hindered older adults’ engagement with digital movement platforms:

1. Fear of injury, which underscored the need for low-impact, customizable movement options tailored to the physical limitations of the elderly;
2. Limited digital literacy, which necessitated an intuitive, simplified UI/UX interface with voice-assisted navigation and guided tutorials;
3. Social isolation, which emphasized the importance of community support features such as peer interaction, hybrid group participation, and social challenges.

These insights shaped the development of Movement Poomasi “*Wello!*”, ensuring that the application addressed both technological and psychosocial barriers through targeted feature design.

### 3.2. System Design and Functional Implementation

The application features a senior-friendly interface with intuitive navigation, high-contrast visuals, large fonts, and voice guidance to accommodate varying levels of digital literacy. The hybrid movement model combines in-person and virtual participation, allowing older adults to select their preferred mode based on mobility, comfort, and social preference. Unlike conventional applications, Movement Poomasi emphasizes peer-led group sessions, social workouts, and recovery features to reduce fatigue and encourage adherence. Communication tools foster engagement through group scheduling, shared progress tracking, and social forums. Feedback from the initial usability trials (Table 3 and Table 4) confirmed improved accessibility and highlighted areas for further refinement.

Specifically, Table 4 consolidates open-ended suggestions from participants, which were categorized into three thematic domains: (1) information, emphasizing the need for exercise guidance tailored to seniors’ physical conditions and transparent reporting of results; (2) education, reflecting strong demand for tutorials, trainer guidelines, and digital literacy training; and (3) social interaction, underscoring the importance of small-group activities, companion participation, and achievement sharing. These insights directly informed the functional expansion of Movement Poomasi, particularly its educational modules, peer-sharing mechanisms, and motivational feedback system. Furthermore, the expressed need for optional features such as location and health status sharing illustrates users’ interest in community-based safety and accountability measures, providing a roadmap for future iterations of the application.

### 3.3. Case Study Benchmarking and Comparative Analysis

A comparative benchmarking analysis was conducted to contextualize the Movement Poomasi application within the broader landscape of digital fitness solutions for older adults. As presented in Figure 1 and Table 1, the majority of existing senior health applications were found to lack three core components: community-based reinforcement mechanisms, hybrid participation frameworks (combining in-person and virtual modes), and interface designs specifically optimized for older users. These platforms predominantly rely on self-directed routines with limited peer interaction and minimal accessibility features tailored to age-related needs.

As shown in Figure 1, the competitive positioning map illustrates that Movement Poomasi uniquely combines social reinforcement, hybrid engagement, and age-tailored interface—an area underrepresented in existing Korean health apps. This strategic combination differentiates the platform from mainstream services that focus solely on individual tracking or automated feedback without social or contextual integration.

Table 1 presents a feature-by-feature comparison of major domestic health and wellness applications, underscoring key functionality gaps such as the lack of community-based feedback and hybrid usability, which Movement Poomasi specifically addresses. This comparison further validates the platform’s differentiated focus and strategic alignment with the real-world needs of aging users in Korea.

In contrast, Movement Poomasi was strategically developed to overcome these limitations by embedding structured social engagement, facilitating seamless transitions between digital and physical modalities, and incorporating a highly accessible, senior-friendly UI/UX architecture. The application’s design decisions were informed by iterative prototyping and expert input, with continuous refinement guided by user feedback.

To further clarify the development trajectory, Figure 2 illustrates the iterative design pipeline of Movement Poomasi “*Wello!*”. The diagram depicts sequential phases—development planning, design, storyboard creation, design selection, technical support, and application development—while the “Revision & Improvement” loop highlights feedback cycles generated by expert review and usability testing. This contextual explanation emphasizes that Movement Poomasi was not created as a linear product but as a continuously refined ecosystem, directly integrating user and expert insights into successive development stages.

**Figure 3:**
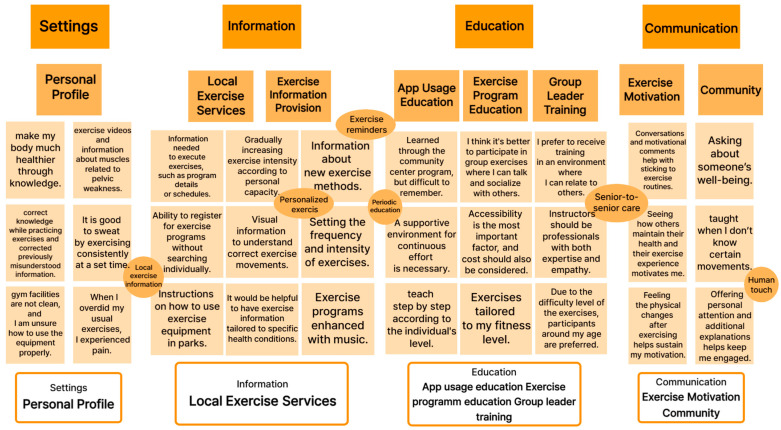
Task flow: interview results—implications and behavioral variables through affinity diagrams.

Furthermore, its alignment with end-user expectations is substantiated by a functional categorization of health and wellness applications (Figure 3, Table 5), which underscores Movement Poomasi’s unique emphasis on mutual assistance, peer collaboration, and education-oriented engagement. Figure 3 further highlights the iterative feedback loop and design refinement process, grounding our interface evolution in direct user insights, particularly in response to challenges identified during pilot testing with older adults.

This multifaceted approach positions the platform not merely as a tool for exercise delivery but as a socially embedded, user-responsive ecosystem tailored to the evolving digital health landscape of aging populations.

### 3.4. Behavioral and Persona Analysis

**Figure 4:**
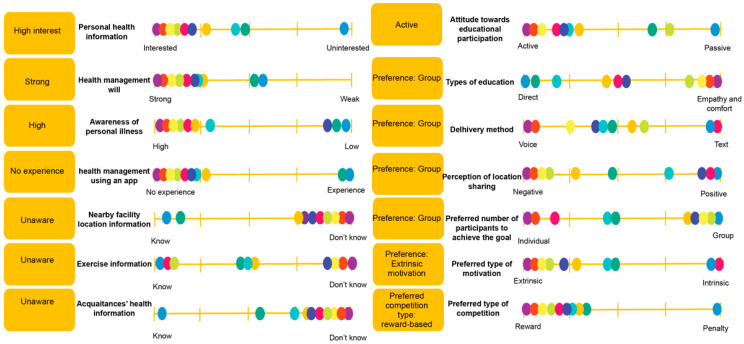
Implications and behavioral variables from interview results. *Implications and behavioral variables from interview results. Colored circles represent individual respondents for visual distinction only and do not correspond to additional variables*.

**Figure 5:**
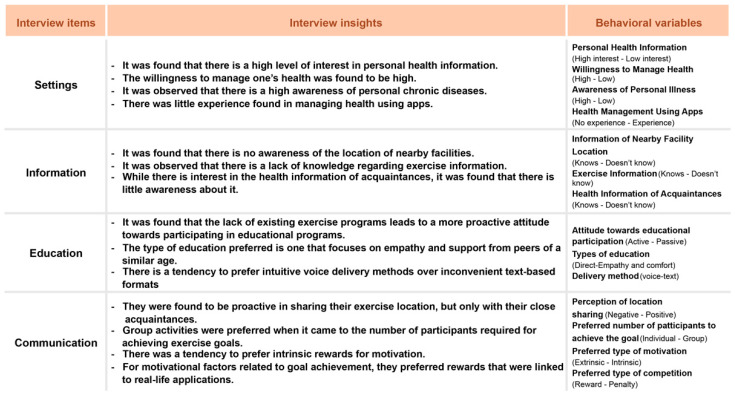
Interview results: Behavioral variables and behavior pattern mapping tasks.

Using persona modeling (Figure 4), two primary user types were identified: movement-active seniors, who are socially motivated and digitally proficient, and reluctant participants, who require simplified interfaces and external encouragement. Behavioral mapping (Figure 5) informed the app’s system refinements, including onboarding tutorials, AI-driven movement suggestions, and interface simplification. This ensured usability for users across the digital literacy spectrum.

### 3.5. Task Mapping and Interface Refinement

**Figure 6:**
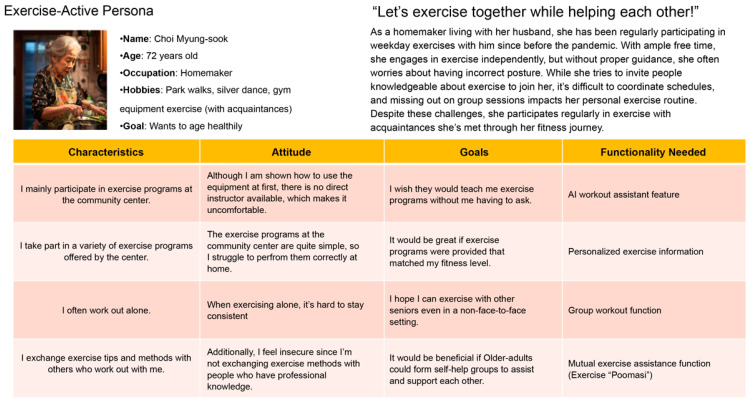
Derivation of personas according to user behavior patterns.

**Figure 7:**
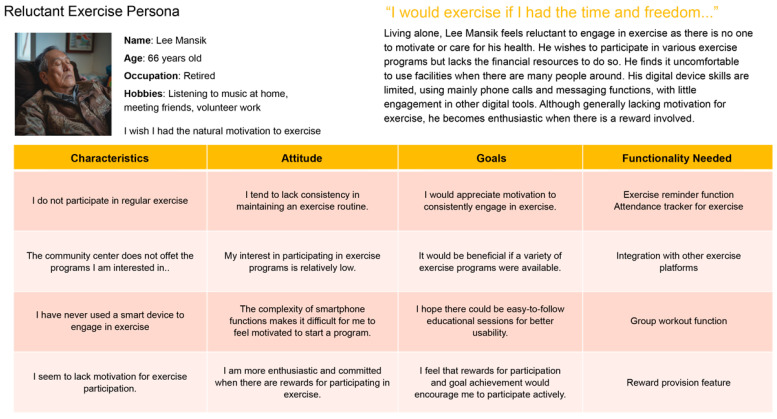
User task status: Movement Poomasi by two functions according to persona function-oriented mapping.

Task mapping and IA design (Table 6) were optimized to match the behavioral characteristics of target users. Guided tutorials, voice-assisted onboarding, and interactive feedback loops were implemented to reduce entry barriers. Social integration features, including community forums and group challenges, were enhanced to support long-term participation. A hybrid activity planner allowed seamless transitions between virtual and in-person exercise environments (Figure 6 and Figure 7).

### 3.6. Usability Testing and Statistical Analysis

Statistical analysis of the 6-week usability trial (*n* = 50) yielded several significant insights into the application’s interface effectiveness and user interaction dynamics. Post-refinement analysis revealed a marked improvement in navigation success rates (*p* = 0.042), confirming the efficacy of the UI/UX modifications implemented during the development cycle.

Analysis of variance (ANOVA) further indicated that tasks related to movement selection and peer interaction required significantly more time to complete (*p* < 0.01), suggesting the presence of cognitive or operational complexity in these interface components. These findings align with the prior literature on digital interaction challenges among older adults and underscore the need for further interface streamlining in cognitively demanding modules.

Regression analysis demonstrated a strong positive correlation between digital literacy and task performance (*r* = 0.68, *p* < 0.05), reinforcing the importance of intuitive onboarding and accessible design elements for users with limited prior experience. While overall satisfaction scores remained high (mean 4.6 ± 0.4), several participants—particularly those with minimal digital exposure—reported usability challenges during complex task flows. Collectively, these results support the strategic incorporation of adaptive onboarding tutorials, simplification of movement selection pathways, and targeted usability enhancements to ensure full inclusivity across varying levels of digital proficiency.

### 3.7. User Satisfaction and Engagement

A total of 15 older adult participants completed two major task-based usability sessions within the Movement Poomasi application. The survey results demonstrated high levels of satisfaction across multiple domains, with the following mean scores (on a 5-point Likert scale):

- Ease of use: 4.6 ± 0.4;
- Encouragement for movement: 4.3 ± 0.5;
- Social connectivity: 4.5 ± 0.3.

A repeated-measures ANOVA revealed a statistically significant improvement in task efficiency over time across the two sessions (*F*(2,28) = 6.32, *p* = 0.005, η^2^ = 0.31), indicating a moderate-to-large effect size. Similarly, paired-sample *t*-tests showed a significant increase in user satisfaction between week 1 and week 6 (t(14) = 2.45, *p* = 0.027), confirming enhanced user experience as engagement deepened.

Correlation analysis confirmed strong associations between perceived ease of use and sustained engagement (ρ = 0.72, *p* = 0.004), as well as between social features and motivation (ρ = 0.53, *p* = 0.038), both statistically significant (*p* < 0.05).

Furthermore, multiple linear regression analysis was conducted to examine predictors of overall user satisfaction. The final model was significant (*F*(3,11) = 4.82, *p* = 0.021, adjusted R^2^ = 0.54), with ease of use (β = 0.46, *p* = 0.019) and social connectivity (β = 0.38, *p* = 0.042) emerging as significant predictors. These findings underscore the critical roles of intuitive design and peer interaction in older adults’ digital health adoption.

Participants with higher prior app usage and more frequent exercise engagement demonstrated shorter task completion times and higher satisfaction scores across UI components.

### 3.8. Data Interpretation

**Figure 8:**
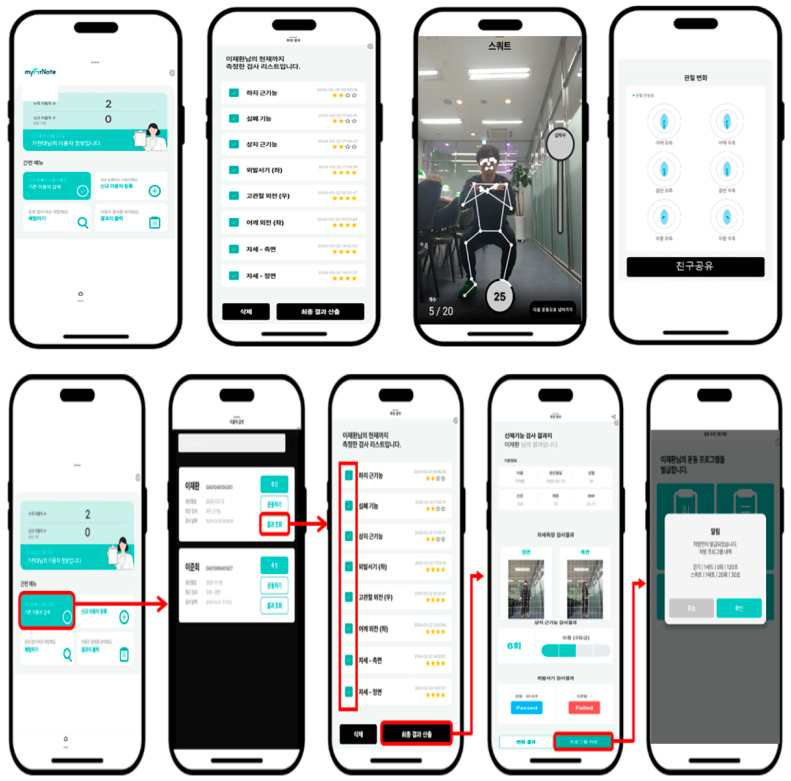
Application main screen flow of Movement Poomasi: (1) Main Dashboard: Displays personal movement summary and access to major functions; (2) Movement Library: Lists categorized movement content (upper body, lower body, cognition, etc.); (3) Movement Execution: Shows real-time feedback during task performance with posture guide; (4) Progress Sharing: Allows users to share their progress with peers; (5) Main Menu: Home, movement info, education, communication tabs; (6) Challenge Participation: Users can join or view peer challenges; (7) Detailed Movement List: Includes difficulty ratings and progress indicators; (8) Movement Evaluation Screen: Allows users to record success/failure of tasks; (9) Feedback Pop-up: Summarizes session results and sends motivational messages.

**Figure 9:**
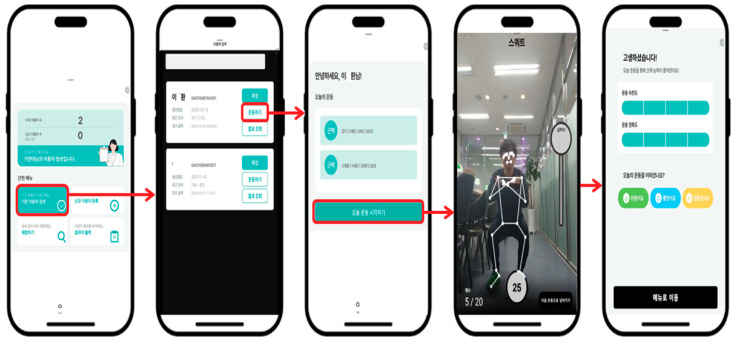
Task evaluation steps *in Movement Poomasi*: (1) Access Dashboard: Tap “Start Evaluation” from the home screen: (2) Select Participant: Choose a registered participant for the task; (3) Begin Task: Initiate real-time guided task with visual posture feedback; (4) Complete Task: Perform and complete the assigned exercise; (5) Submit Evaluation: Self-evaluate task with options (Passed/Failed/Retry).

Table 7, Table 8, Table 9, Table 10 and Table 11 summarize key quantitative outcomes, including usability scores, task efficiency, and satisfaction levels, providing a comprehensive overview of how older adults engaged with Movement Poomasi “*Wello!*”. Figure 8 and Figure 9 illustrate the representative task flow and user interface interactions of the application. Although the interface screens are presented in Korean—reflecting the application’s local development and distribution context—each image has been carefully annotated with numeric labels and accompanied by detailed English captions to ensure clarity for international readers. These annotations provide functional explanations of each screen component and facilitate accurate interpretation of the user interaction flow despite the language of the original interface.

Table 7 highlights baseline usability metrics, showing that while dashboard navigation and movement execution achieved relatively higher success rates, more cognitively demanding tasks—such as movement selection and peer interaction—showed lower initial completion accuracy. Table 8 focuses on task efficiency, indicating that participants aged ≥75 required significantly more time for menu navigation, suggesting the need for further streamlining for the oldest subgroup. Table 9 demonstrates the central role of peer-based engagement, as tasks involving social features (e.g., challenge participation, progress sharing) achieved the highest success rates and satisfaction levels. Table 10 presents error rates and recovery pathways, showing frequent difficulties with movement filtering, but also confirming that onboarding tutorials and simplified cues substantially improved recovery success. Finally, Table 11 synthesizes overall user satisfaction, demonstrating consistently high ratings for ease of use, motivational feedback, and social connectivity. Notably, participants with higher baseline digital literacy and regular exercise experience reported shorter task completion times and greater satisfaction, underscoring the importance of prior familiarity in predicting long-term engagement.

Taken together, these interpretative summaries illustrate the progressive refinement of Movement Poomasi “*Wello!*”, from identifying baseline barriers (Table 7) to enhancing efficiency (Table 8), leveraging social reinforcement (Table 9), improving error recovery (Table 10), and consolidating satisfaction outcomes (Table 11). Sequentially, these findings confirm that Movement Poomasi effectively reduced usability barriers, maximized engagement through social reinforcement, and adapted to diverse digital literacy levels among older adults.

To ensure transparency and reproducibility, a structured summary of all statistical analyses—including test statistics, degrees of freedom, *p*-values, effect sizes, and confidence intervals—is provided in Table 12 (summary of statistical analyses). This APA 7th-compliant consolidated table not only strengthens clarity and scientific rigor but also ensures alignment with international reporting standards, thereby enhancing the global relevance and credibility of the study’s findings.

## 4. Discussion

This study developed and evaluated Movement Poomasi, a digital healthcare application aimed at enhancing physical activity, social interaction, and accessibility for older adults. By combining adaptive programming, hybrid participation models, and peer motivation tools, the app promoted long-term engagement. These findings align with those of prior research emphasizing the role of digital interventions in promoting active aging but extend this work by demonstrating the benefits of community-integrated design and hybrid structures.

Technology acceptance varied significantly across participants, consistent with earlier studies on the digital divide among older adults [32,33,34]. Usability testing confirmed that digital literacy was positively correlated with task success (*r* = 0.68, *p* = 0.031), revealing that lower proficiency increased task time and difficulty. However, this study highlighted that onboarding guidance and intuitive interface design significantly improved performance. Post-refinement *t*-tests showed a significant usability improvement (*p* = 0.042), supporting UI/UX modifications as a key strategy for inclusion.

Despite improvements, certain tasks, especially movement selection and peer engagement, remained complex, as confirmed by the ANOVA results (*p* = 0.007). These findings echo the prior literature on cognitive overload in digital interfaces for seniors [34] and underscore the need for further simplification and guided assistance features. They also align with perspectives on systemic social innovation, which emphasize co-creating sustainable futures for both individuals and communities [35].

Digital inequality has become a notable structural barrier. Consistent with previous research [36,37], users from lower-income or rural backgrounds face challenges such as limited device access or connectivity. Although the hybrid model offers alternative participation modes, it only partially addresses systemic disparities. Nonetheless, this approach supports Czaja et al.’s [38] recommendation to integrate digital and traditional services to enhance their reach. Furthermore, social engagement features showed a positive correlation with motivation (ρ = 0.53, *p* = 0.048), reinforcing the value of peer support in sustaining movement [39].

Within the broader framework of digital inequality, the sociocultural context of South Korea reveals entrenched gender norms that further exacerbate disparities in digital engagement. Older men, often positioned as household decision-makers, tend to exert greater control over the adoption and use of technology, which can inadvertently restrict older women’s access to and independent utilization of digital tools—particularly in areas perceived as non-essential, such as health-related digital leisure or exercise applications. Although older women demonstrate relatively high engagement with interpersonal communication tools such as messaging platforms or social networking services, their participation in structured digital leisure domains, including virtual fitness programs or online learning environments, remains markedly limited. This gendered disparity appears to stem from a confluence of factors, including lower digital self-efficacy, entrenched caregiving responsibilities, and limited exposure to diverse digital media. The prevalence of male-headed households in older populations may further centralize control of technological resources, deepening the gender gap. Addressing these inequities requires gender-responsive digital inclusion strategies that equip older women with the skills, confidence, and autonomy to navigate digital ecosystems effectively. Practical approaches include hands-on intergenerational training, peer mentorship programs, and community-based educational models, alongside policy interventions such as subsidized digital access and targeted public campaigns.

The app also addresses age-related cognitive and motor challenges. Participants cited navigation and comprehension difficulties due to declining memory or dexterity, consistent with earlier research [33,40]. However, high satisfaction scores for ease of use (4.6 ± 0.4) indicated that features such as high-contrast visuals, voice guidance, and simplified layouts mitigated these issues. However, further cognitive load reduction through AI-driven onboarding and predictive assistance is warranted.

Finally, the broader implications suggest that Movement Poomasi has the potential to become a scalable solution for the aging global population. By supporting multilingual content, cultural adaptability, and localized health integration, the platform can serve diverse older adults worldwide. Future research should include emotional well-being assessments and longitudinal evaluations to better capture the full impact of digital health interventions on the physical and mental resilience of older adults [41].

### 4.1. Key Findings and Impact

The app’s tailored programming, hybrid structure, and peer motivation features substantially improved physical activity adherence and social connectivity among older adults. Participants benefited from personalized feedback, virtual and in-person challenges, and community-led initiatives that fostered both individual engagement and collective motivation. Compared to existing fitness applications, Movement Poomasi demonstrated greater accessibility, deeper social integration, and stronger alignment with the physical and cognitive needs of older adults.

The platform effectively addresses common challenges in senior fitness technology by offering a user-friendly, socially integrated solution that combines the flexibility of a hybrid participation model with personalized content and continuous UI adjustments. This multifaceted approach creates a well-rounded intervention that is adaptable to older adults with diverse requirements and preferences.

However, task complexity remains an obstacle, particularly for users with low digital skills. Key interface areas—such as movement selection and group communication—require further optimization to ensure full inclusivity. Targeted refinement of these elements will be critical to maximizing accessibility and sustaining long-term engagement across varying levels of digital literacy.

### 4.2. Limitations and Strengths of the Study

This study possesses notable strengths, including its hybrid engagement model, iterative development process, and the integration of both qualitative and quantitative evaluation methods. The user-centered design actively involved older adults and multidisciplinary experts, ensuring that the platform was both scientifically grounded and practically relevant for aging populations.

Nonetheless, several limitations must be acknowledged:

1. Sample Bias: The majority of the participants had moderate levels of digital exposure, which may limit the generalizability of the findings to digitally marginalized or technology-averse older adults. Future studies should include participants with minimal or no prior technical experience to more accurately reflect the digital diversity within aging populations.
2. Self-Reported Measures: Usability and engagement metrics relied primarily on self-reported data, which can be susceptible to recall bias or social desirability effects. Integrating objective behavioral tracking and backend analytics in future studies would enhance data reliability and precision.
3. Cultural Context: As this study was conducted within the South Korean healthcare and sociocultural context, the applicability of the results to other regions may be limited. Cross-cultural validation is needed to assess adaptability in healthcare environments with differing technological infrastructures and aging paradigms.
4. Duration of Evaluation: A key limitation lies in the relatively short duration of the usability trial. While the 6-week implementation allowed for targeted feedback and interface refinement, it was insufficient to capture long-term behavioral adaptation, sustained engagement, or app retention. Older adults often require prolonged exposure to establish digital habits, particularly when cognitive or motor declines are present. From a development standpoint, this limited window may have masked latent barriers such as motivational fatigue, seasonal disruptions, or shifting preferences over time. Longer-term studies with follow-up assessments are necessary to validate the durability and ecological validity of the intervention in real-world aging trajectories. The application has continued iterative testing and refinement until May 2025, and it is scheduled for re-launch under the name *Wello!* in August 2025.

Despite these limitations, this study provides compelling evidence that digital health tools, when designed with intentional, user-sensitive strategies, can be both accessible and engaging for older adults. The findings offer meaningful insights for the development of inclusive, technology-based interventions that support healthy aging in diverse populations.

This study reinforces the notion that long-term adherence is closely associated with intuitive design, community support, and flexible participation options. Personalized movement tracking, simplified navigation, and social reinforcement create a sustainable engagement loop. Future iterations should prioritize refining onboarding, expanding AI-generated exercise plans, and developing mentorship features.

### 4.3. Future Directions for Development

Based on the current results, future enhancements will focus on the following:

- Onboarding Improvements: Adding real-time tutorials, step-by-step guidance, and AI-driven support for first-time users.
- Gamified Engagement: Including rewards, progress milestones, and personalized goal-setting to boost motivation.
- Community Integration: Expanding partnerships with senior centers and healthcare providers to connect digital use with local wellness resources.

Through these refinements, Movement Poomasi can evolve into a comprehensive platform that supports socially connected active aging in both digital and physical spaces.

## 5. Conclusions

This study provides a comprehensive analysis of the usability, accessibility, and effectiveness of digital healthcare applications for older adults, using Movement Poomasi as a case study. The findings confirm that technology adoption among older adults varies widely, influenced by factors such as digital literacy, cognitive and physical limitations, and prior exposure to smart technology. However, the study demonstrated that structured design interventions, hybrid engagement models, and social reinforcement strategies can significantly enhance usability, participation, and long-term adherence to exercise programs.

The findings underscore the necessity of a multidimensional approach to digital health adoption, emphasizing the interplay among usability-focused design, social integration, and global scalability. The hybrid nature of Movement Poomasi, which allows for both virtual and in-person exercise engagement, presents a model for bridging the digital divide and accommodating older adults with diverse mobility and technological needs. This approach aligns with global aging trends and highlights the potential of digital healthcare applications to go beyond physical health improvements by fostering mental well-being, social connectedness, and overall quality of life in older adults.

Our findings further underscore the feasibility and acceptability of a culturally grounded, digitally augmented intervention for older adults. The model’s adaptability to different cultural contexts—particularly those with similar communal traditions or hybrid care needs—offers promising avenues for international application. Future policy and public health initiatives should consider localized digital inclusion strategies grounded in cultural familiarity and peer-based motivation to ensure sustainable engagement and long-term health impact among aging populations.

## Figures and Tables

**Table 1:** Functional comparison of representative Korean health and exercise management applications (preliminary market scan).

	Dajim	Health Talk	Today’s Health	Plan Fit	Samsung Health	Insight for Movement Poomasi
Exercise facilities, programs, and trainers search	○	X	X	○	X	Limited guidance for seniors; Movement Poomasi offers curated content for age-appropriate routines
Schedule management	○	○	○	○	○	Common feature; Movement Poomasi includes reminder-based scheduling with hybrid integration
Health goal-setting and -recording	○	○	○	○	○	Standard across apps; Movement Poomasi links goal-setting to peer challenges
Peer exercise records comparison	X	○	X	○	○	Mostly absent; Movement Poomasi enables structured social sharing
Interactive educational content	X	X	X	X	X	Partially present; Movement Poomasi provides task-oriented video guidance
Leader/facilitator support	X	X	X	X	X	Unique to Movement Poomasi, with peer and guide options
App usage education	X	X	○	X	○	Weak overall; Movement Poomasi includes onboarding walkthrough and voice assistant
Self-help exercise groups operation	X	X	X	○	X	Movement Poomasi enables autonomous group challenges
Achievement sharing	X	○	○	X	○	Often one-way; Movement Poomasi supports reciprocal social encouragement

*Note. ○ = Feature fully supported; X = Feature not supported.*

**Table 2:** Composition of the exercise program.

Menu	Content		
Home Training	Warm-up/cool-down training	Walking, stretching, and joint mobilization exercise	
	ACPT-Fascial Circulation Exercise	Rhythmic fascia flow movements, breath-synchronized stretches	
	Main training	Circuit training	Whole-body low-impact circuit, cardiovascular endurance, flexibility exercise
		Targeted training	Arm strengthening, core stability, leg balance training
		Sensory feedback modules	Proprioceptive tapping, body scan, breathwork

**Table 3:** Deriving implications related to exercise types for the elderly.

Type	Before COVID-19	After COVID-19	Problem Recognized	Suggestion
Active Group	Interest in various types of exercise, engaging in physical activity for at least 1 h, 3 times a week. High concern for health.	Exercise participation was either maintained or slightly increased.	Curiosity about different exercise methods. The enjoyment of exercising together.	Provide exercise reminders and competitive features.
Passive Group	Walking or using park exercise equipment, exercising less than 1 h, 2 times a week or less. Low concern for health.	Decrease in exercise participation due to the closure of facilities.	Habitual participation. Need for information that supports consistent participation.	Offer information on local sports facilities, equipment, and instructors.
Psychological Support	Exercise helps with stress relief and mood improvement.	Perception of lethargy and loneliness due to lack of physical activity.	Content necessary to sustain exercise in non-face-to-face situations.	Deliver interactive, non-face-to-face exercise content.
Companions and Supporters	The presence of a companion enhances the effectiveness of exercise, while the absence of a companion disrupts exercise routines.	Continued exercise with support from family and friends.	Social support content from exercise partners and peers.	Enable communication through community features.
Motivation and Interest in Exercise	Participating in exercise to manage disease levels and alleviate boredom.	Reduced interest in exercise due to environments that make participation challenging.	Need for personalized exercise programs.	Provide personalized exercise programs.
Use of Digital Devices for Exercise	Sharing exercise information with those who exercise together.	Active participation in digital exercise programs provided by welfare centers.	Provision of educational programs to utilize app functions.	Offer digital literacy education programs.

**Table 4:** Core open-ended user suggestions and derived keywords.

Suggestions	Core Keywords
▪ Provision of exercise information tailored to the physical condition and fitness level of older adults ▪ Encouraging and motivational messages should be provided during exercise ▪ Goal-setting and comparison features with peers’ records should be available ▪ Reward mechanisms (badges, points, or certificates) should be provided upon goal achievement ▪ Recommendation services based on peer user data should be integrated ▪ Music playback functionality during exercise ▪ Clear, transparent feedback and progress reports on exercise results ▪ Information on local programs, certified trainers, and facilities should be provided ▪ Explanations about exercise benefits and physiological effects should be included	Information
▪ Interactive education modules designed for older adults ▪ Guideline-based training for exercise programs (targeted at trainers and caregivers) ▪ Digital literacy training and in-app tutorials for users ▪ Ethical guidelines for trainers and community facilitators	Education
▪ Small-group participation features to promote peer support ▪ Companion-based participation options (family, friends, caregivers) ▪ Social sharing of exercise photos, videos, and achievements ▪ Tools to organize, manage, and sustain self-help exercise groups	Social Interaction
▼	
When older adults engage in exercise, it is essential to provide an interactive service framework that integrates information, education, and social communication features. This should include personalized exercise data, digital literacy support, and peer-engagement mechanisms to ensure both accessibility and motivation. Additionally, as an optional feature based on user agreement, the system should incorporate a dedicated UI that enables location sharing, health status monitoring, and streamlined group management, thereby enhancing both individual safety and collective participation.	

*▼: It indicates that the results presented above lead to the outcomes shown below.*

**Table 5:** Summary of major tasks and functions by key keywords related to new exercise and healthcare app services.

Situation	Task	Related Function	APP Type	Service Type
When an Older adult wants to participate in remote (or non-face-to-face) exercise	Local exercise information (information)	Location-based integrated search function	Dajim, Plan Fit	Self-Care
	Personalized exercise information (information)	Health status check and exercise recommendation function	Health Talk Talk, Today’s Health, Plan Fit, Samsung Health	Self-Care
	Interactive educational content (education)	Exercise method education	Health Talk Talk, Today’s Health, Plan Fit, Samsung Health	Mutual Assistance Care
		App usage education	Today’s Health	
		Group leader training	-	
		IT exercise instructor training	-	
	Small-group exercise participation content (communication)	Small-group health goals and exercise records		Mutual Assistance Care

**Table 6:** Information architecture (IA) reflecting integrated user tasks.

1 Depth	2 Depth	3 Depth	4 Depth	Main Features
Home (Main page)	My Health Profile	Information Integration	-	Settings
	Today’s Exercise	Educational Integration	-	Check Exercise Schedule
	Friends List	Communication Sharing Integration	-	Exercise Sharing
Information Sharing	Local Services	Local Welfare Centers	Exercise Programs	Search/Apply
			Program Capacity	Search
	Games	Exercise and Music	Warm-Up—Cardio Cool-Down	Gamification
		Senior Dance		
	Personalized Exercises	Senior Surveys	Personalized Exercise Information	Personalized Information Provision
	Exercise Results	Calories	-	Exercise Results Review
		Exercise Duration		
Education Sharing	App Usage Training	App Usage Guide	APP Q&A	App User Guide
	Exercise Training	Cardio Exercise	Individual/Group Exercise	AI Motion Exercise Training
		Strength Training		
	Exercise Platform	Engage	Metaverse Exercise	Exercise Education
		Dipda	GX Exercise Program	GX Exercise
Communication Sharing	Exercise Motivation	Exercise Notifications	Voice/Vibration Guidance	Notification
		Exercise Attendance Tracker	-	Check/Reserve
		Exercise Ranking System	Points by Ranking	Exercise Management
My Profile	View Various Information Details	Medication and Prescription History Integration		Personal Information Terms/Policy Guidance

**Table 7:** Usability testing metrics.

1 Depth	Content	Key Features
Efficiency	How many steps were completed during task performance? Which steps were the most difficult?	Success rate
	How much time does task performance require?	Time taken
Satisfaction	What is the user satisfaction level after task completion?	User satisfaction
Ease of Use	Is the program easy to operate?	Ease of operation
	Are menus, buttons, and options easy to locate?	Visibility
	Is reading and viewing text or images comfortable?	Intuitiveness
Educational Ease	The learning objectives are clear.	Goal clarify
	The method of presenting exercise education captures attention.	Enjoyment
	The method of delivering exercise education motivates participation.	Motivation
Information Usefulness	The provided information is practical and useful for real-life exercises.	Usefulness
	The quantity of provided information is sufficient.	Information quantity
	New insights or knowledge were gained.	Novelty

**Table 8:** Individual task success rates and task completion times.

Participant	Gender	Age	Exercise Frequency	Exercise Duration (Mins)	Participation Level
U1	F	64	3–4 times per week	30	Active
U2	F	72	1–2 times per week	90	Passive
U3	F	72	3–4 times per week	60	Active
U4	F	68	1–2 times per week	60	Passive
U5	F	75	More than 5 times per week	60	Active
U6	F	65	1–2 times per week	30	Passive
U7	F	68	3–4 times per week	30	Passive
U8	F	64	1–2 times per week	30	Passive
U9	M	72	More than 5 times per week	90	Active
U10	M	67	1–2 times per week	60	Active
U11	M	77	More than 5 times per week	90	Active
U12	M	64	1–2 times per week	30	Passive
U13	M	67	3–4 times per week	30	Active
U14	M	65	1–2 times per week	30	Active
U15	M	70	3–4 times per week	60	Active

**Table 9:** Overview of initial usability evaluation progress.

Participant	Task 1: Completed Steps (1–5)	Task 1: Time Taken (s)	Task 2: Completed Steps (1–5)	Task 2: Time Taken (s)
U1	4	30	5	27
U2	3	41	5	29
U3	5	28	5	27
U4	5	29	5	28
U5	5	27	5	28
U6	4	31	5	29
U7	4	32	5	28
U8	5	27	4	31
U9	4	32	5	27
U10	4	32	4	33
U11	5	27	5	25
U12	4	34	5	28
U13	3	42	5	29
U14	4	32	5	29
U15	5	27	5	28

**Table 10:** Efficiency analysis results for Tasks 1 and 2.

Task	Task Success Rate (%)	Task Time (s)	Efficiency (Average)	Remarks
1	85.3%	31.6	2.7	Simplification of selection needed
2	97.3%	28.4	3.4	Confirmation of exercise information needed

**Table 11:** Overview of final usability evaluation progress.

Participant	Task 1 Satisfaction with Related Main Functions (1–5 Points)					Task 1 Satisfaction with Related UI (1–5 Points)					Task 2 Satisfaction with Related Main Functions (1–5 Points))					Task 2 Satisfaction with Related UI (1–5 Points)				
1–5 Stage	1	2	3	4	5	1	2	3	4	5	1	2	3	4	5	1	2	3	4	5
U1	2	5	4	5	4	3	5	5	5	5	2	5	5	5	5	5	3	5	5	4
U2	4	5	4	5	5	5	5	4	5	5	4	5	5	3	5	5	5	5	3	5
U3	3	5	4	4	3	5	5	3	5	4	5	4	5	5	4	3	3	5	4	5
U4	4	4	5	5	4	4	5	2	4	5	5	5	5	4	5	5	5	5	5	5
U5	5	5	4	5	5	5	4	4	5	5	5	4	4	5	5	3	5	5	4	4
U6	5	4	4	5	3	4	4	4	4	4	4	5	3	3	3	2	5	5	4	4
U7	5	4	5	5	4	4	5	5	5	5	5	4	5	5	5	5	4	4	5	5
U8	5	5	5	5	5	5	5	4	5	5	5	5	5	5	5	5	5	5	5	5
U9	4	5	2	5	2	2	4	5	5	3	5	4	4	3	5	5	4	5	4	5
U10	5	5	4	5	5	4	4	2	4	5	4	4	3	5	4	5	5	4	3	5
U11	4	5	5	5	5	5	3	5	5	5	5	5	4	5	5	4	5	4	5	4
U12	5	5	5	5	4	4	5	3	4	5	4	5	5	5	4	4	4	4	2	5
U13	5	5	5	5	5	5	5	5	5	5	5	5	5	5	5	5	5	5	5	5
U14	5	5	4	5	4	4	4	2	5	5	5	5	4	5	5	5	5	5	5	4
U15	4	5	5	4	5	5	5	5	5	5	5	4	5	4	5	5	5	5	5	5
Satisfaction Average	4.33	4.80	4.33	4.87	4.20	4.27	4.53	3.87	4.73	4.73	4.53	4.60	4.47	4.47	4.67	4.40	4.53	4.73	4.27	4.67

**Table 12:** Summary of statistical analyses.

Test	Variables/Comparison	df	Test Statistic	*p*-Value	Effect Size	95% CI (Where Applicable)
ANOVA	Task efficiency over time (Session 1–2–3)	*F*(2,28)	6.32	0.005	η^2^ = 0.31	[0.12, 0.48]
Paired *t*-test	User satisfaction (week 1 vs. week 6)	t(14)	2.45	0.027	d = 0.63	[0.08, 1.12]
Correlation	Ease of use ↔ sustained engagement	–	*r* = 0.72	0.004	ρ = 0.72	–
Correlation	Social features ↔ motivation	–	*r* = 0.53	0.038	ρ = 0.53	–
Regression	Predictors of overall user satisfaction (ease of use, social connectivity, prior app use)	*F*(3,11)	4.82	0.021	Adj. R^2^ = 0.54	–
Regression (β)	Ease of use	–	β = 0.46	0.019	–	–
Regression (β)	Social connectivity	–	β = 0.38	0.042	–	–
Chi-squared test	Success rate after UI refinement	χ^2^(1)	4.12	0.042	φ = 0.29	–
ANOVA (task completion)	Movement selection vs. peer interaction modules	*F*(1,49)	7.21	<0.01	η^2^ = 0.22	–

Note All *p*-values are two-tailed. Effect sizes are reported as η^2^ (ANOVA), Cohen’s *d* (*t*-test), *r* (correlation), φ (chi-squared), and standardized β (regression coefficients); 95% confidence intervals are reported where applicable. Interpretation of effect sizes follows Cohen’s (1988) conventions: small (*d* = 0.2, η^2^ = 0.01, *r* = 0.10), medium (*d* = 0.5, η^2^ = 0.06, *r* = 0.30), and large (*d* = 0.8, η^2^ = 0.14, *r* = 0.50).

## Data Availability

The data supporting the findings of this study are available upon reasonable request from the corresponding author. Due to ethical and privacy considerations, access to the data may require approval and adherence to institutional guidelines. If applicable, anonymized datasets can be shared for academic and research purposes, subject to compliance with relevant data protection regulations. Any additional materials, including analysis code, may also be provided upon request.

## Abbreviations

AI: artificial intelligence ANOVA: analysis of variance IA: information architecture UI/UX: user interface/user experience.
